# Editorial

**Published:** 1867-05

**Authors:** 


### Editorial.

Chicago Medical Society—Annual Meeting.—The regular annual meeting of the Chicago Medical Society was held in the Court House, on the evening of the 5th of April, 1867. The meeting was well attended, and the election of officers for the ensuing year, resulted as follows: President, Dr. J. P. Ross; Vice-President, Dr. John Reid; Secretary, Dr. H. M. Lyman.

*Sanitary Committee.*—Drs. N. S. Davis, R. G. Bogue, and R. M. Lackey.

*Censors.*—Drs. E. L. Holmes, Thos. Bevan, John Reid.

*Committee on Ethics.*—Drs. S. Wickersham, G. Paoli, T. D. Fitch.

*Delegates to the American Medical Association.*—Drs. Macalister, Paoli, Wickersham, Reid, Blake, Bogue, Lackey, Fitch, and Fisher.

*Delegates to the Illinois State Medical Society.*—Drs. Davis, Trimble, Fisher, Macalister, Bogue, Holmes, Lackey, Fitch, Hatch, Hildreth, Nelson, Loverin, Tucker, Reid, Blake, Quales, Fenn.

A vote of thanks was unanimously tendered to the retiring officers for the faithful and prompt manner in which they had discharged their respective duties during the past year. After the transaction of some miscellaneous business, the society adjourned.

---

Chicago Medical College Appointments.—During the past winter, Prof. F. Mahla, who had ably filled the two Chairs of Chemistry from the first organization of the College, tendered his resignation, in consequence of the pressure of other business. The places thus made vacant have been filled by the appointment of ——— Davies, Prof. of Chemistry and Physics in the Lawrence University, at Appleton, Wisconsin. Dr. Davies is an enthusiastic cultivator of the natural sciences, and will bring to his new position a high reputation, founded on several years' experience as a practical teacher. Daniel Nelson, M.D., who has given the course of instruction on Physiology and Histology, during the past lecture term, in a manner highly satisfactory to the Faculty and Students, has now been appointed to the full Professorship in that department. Prof. H. A. JOHNSON, who felt compelled last year to resign the Chair of General Pathology and Public Hygiene, on account of ill-health, has again accepted a Professorship, to which has been assigned the full consideration of Diseases of the Chest. There is an excellent class of students in attendance on the Spring and Summer Course of Instruction, now in progress, and the College is steadily advancing in its career of usefulness and prosperity.

---

ILLINOIS STATE MEDICAL SOCIETY.—The next Annual Meeting of the Illinois State Medical Society, will be held in the Hall of the House of Representatives, at Springfield, commencing on the first Tuesday in *June*, at 10 o'clock, A. M.

We are authorized, by the Committee of Arrangements, to state, that the Chicago, Alton, and St. Louis, and the Toledo, Wabash, and Great Western Railroads, will pass Members of the Society to and from the Meeting, over these roads, for one fare and one-fifth. It is also expected that the Illinois Central will do the same.

N. S. DAVIS,

*Per. Sec. Ill. State Med. Society.*

*Chicago, April 15, 1867.*

---

We take pleasure in calling the attention of our readers to the following notices:—

TO PHYSICIANS.—At the request of several members of the profession, Dr. Horatio R. Storer will deliver a private course of twelve lectures, upon the Treatment of the Surgical Diseases of Women, during the first fortnight of June, at his rooms in Boston. Gentlemen attending the course, will be required to show their diplomas. Fee $50.

*Hotel Pelham, Boston, March 29, 1867.*

CHARITABLE EYE AND EAR CLINIC, NO. 305 STATE, COR. VAN BUREN ST.—Trustees:

Hon. Lyman Trumbull, President.
C. T. Bowen, Esq., Secretary and Treasurer.
W. E. Doggett, Esq.
C. H. Holden, Esq.
Jos. G. Hildreth, M.D. Attending Surgeon.

This institution is open daily, at 12 o'clock, for the treatment of the poor of the Northwest.

Clinical instruction will be given during the lecture season.

---

INDIANA STATE MEDICAL SOCIETY.—The next regular meeting of the Indiana State Medical Society will be held at Indianapolis, Ind., on Tuesday, May 21st, 1867. We hope all our readers in that State will remember the day, and see that their respective localities are well represented.

---

CHICAGO MEDICAL JOURNAL.—This periodical has again changed hands. Drs. HOLMES, LYMAN, and LACKEY have vacated the editorial chair, and given place to Dr. J. ADAMS ALLEN, Professor of Principles and Practice of Medicine in Rush Medical College. The retiring editors were men of talent, industry, and integrity, and their sudden displacement will be regretted by many. Their successor is well known to the readers of that journal, he having occupied the same position in connection with the late Prof. BRAINARD, three or four years since.

---

MONEY RECEIPTS FROM FEB. 21ST TO MAR. 22D.—Drs. R. D. Cogswell, Nauvoo, Ill., $3, E. Robbins, Pontoosuc, Ill., 3, P. G. Kelsey, Fort Wayne, Ind., 5, W. L. Krieder, Prairie City, Ill, 3, Wm. Hoyne, Monmouth, Ill., 3, A. C. Corr, Carlinville, Ill., 3.00, G. Bradley, Waverly, Ill., 3, D. La Count, Chilton, Wis., 8, G. A. Bradwell, Prophetstown, Ill., 3, H. C. Lester, Newcomer, Ind., 3, S. D. Mercer, Omaha, Neb., 3, M. A. McClellan, Canton, Ill., 3, St. Joseph Medical Society, St. Joseph, Mo., 3, John Griffin, Newton, Ill., 3, M. S. Wilson, Griggsville, Ill., 3, J. H. Hollister, Chicago, Ill., 5, Herbert Harris, Wheeler, Ind., 3., E. V. Doles, Chicago, Ill., 3.

### Mortality Report for the Month of February:—

### CAUSES OF DEATH.

| | | | |
|---|---|---|---|
| Accidents, | 2 | Fever, Scarlet, | 10 |
| Abscess, | 1 | Fever, Typhoid, | 5 |
| Bronchitis, | 1 | Fever, Childbed, | 2 |
| Cancer, | 2 | Fever, Typhus, | 1 |
| Colds, | 6 | Hydrocephalus, | 10 |
| Croup, | 8 | Inflammation of Bowels, | 7 |
| Consumption, | 40 | Inflammation of Brain, | 3 |
| Convulsions, | 43 | Inflammation of Lungs, | 11 |
| Childbed, | 4 | Inflammation of Throat, | 1 |
| Congestion of Brain, | 1 | Inflammation, not stated, | 2 |
| Congestion of Lungs, | 7 | Killed, | 1 |
| Delirium Tremens, | 1 | Marasmus, | 1 |
| Drowned, | 1 | Measles, | 1 |
| Decline, | 1 | Old Age, | 9 |
| Diphtheria, | 5 | Poisoning, | 1 |
| Dropsy, | 7 | Paralysis, | 3 |
| Disease of Brain, | 1 | Pneumonia, | 2 |
| Disease of Bowels, | 1 | Rheumatism, | 1 |
| Disease of Heart, | 4 | Suicide, | 1 |
| Disease of Liver, | 2 | Stillborn, | 8 |
| Disease of Lungs, | 5 | Spasms, | 4 |
| Disease of Hip, | 1 | Small Pox, | 3 |
| Erysipelas, | 1 | Teething, | 12 |
| Fever, Congestive, | 6 | Whooping-Cough, | 10 |
| Fever, Remittent, | 3 | Ulcer, | 1 |
| Fever, Spotted, | 1 | Unknown, | 15 |

Total, 280

Total number last year for the month of March, 254

Increase, 26

### DIVISIONS OF THE CITY.

North, 67 | South, 90 | West, 123 | Total, 280

Total number during the month of March, 280

Total number during the month of February, 255

Increase, 25

Ages of the Deceased.—Under 5 years, 143; over 5 and under 10 years, 16; over 10 and under 20, 16; over 20 and under 30, 29; over 30 and under 40, 27; over 40 and under 50, 17; over 50 and under 60, 7; over 60 and under 70, 9; over 70 and under 80, 10; over 80 and under 90, 2; over 100, 2; unknown, 2. Total, 280.

### NATIVITIES.

| | | | | | |
|---|---|---|---|---|---|
| Chicago, | 151 | France, | 1 | Sweden, | 2 |
| Other States, | 48 | Germany, | 38 | Scotland, | 3 |
| Bohemian, | 1 | Holland, | 1 | Unknown, | 1 |
| Canada, | 4 | Ireland, | 25 | | |
| England, | 2 | Norway, | 3 | Total, | 280 |

---

The attention of the members of the Massachusetts Medical Society is respectfully called to the following communications from the *Boston Medical and Surgical Journal*:—

*Treatment of Acute Rheumatism.* Messrs. Editors,—Having for a year past used what I consider a new remedy for rheumatism, and with better success than from any other remedy, I consider it proper to ask the profession to make a trial of it. It is the syrup of lime, made according to Trousseau's prescription, as found in Parrish's Pharmacy. I have used it, according to the severity of the case and the age of the patient, in the dose of ten (10) drops to forty-five (45) drops, and repeated in from two (2) to six (6) hours, as symptoms have seemed to demand. In but one (1) case has any opiate been required from the beginning. Two (2) cases were complicated with Bright's disease, as indicated by the great abundance of albumen, and the casts, as seen in the urine. In one of these the albuminuria entirely dissappeared, and in the other it has been largely diminished.

There has been no constipation, but generally looseness of the bowels, after a couple of days' treatment.

The medicine is best taken in unskimmed milk, in quantity from a tablespoouful to four (4) ounces, according to the size of the dose of syrup.

Hoping that other members of the profession may meet with the success which I think I have had, I am very truly yours,

*Boston, Eebruary 23, 1867.* CHAS. E. BUCKINGHAM.

For the information of our readers, we copy from Parrish's Pharmacy the prescription alluded to:—

"CALK SACCHARATUM, SYRUPUS CALCIS.—Trousseau used the following proportions for producing a solution of lime by the aid of sugar:—I part of slaked lime, 10 parts water, and 100 parts syrup are boiled together for a few minutes, strained and diluted with four times the weight of simple syrup.

"This syrup has an alkaline taste and reaction, and is the solution of a chemical compound of sugar and lime. It is used for the same purposes as lime water, but on account of its causticity it is necessary to dilute it considerably. It is given to children in the quantity of twenty or thirty grains during the day; adults take from two to three drachms during the same time."

---

*City and Country Population of England.*—According to the Registrar-General, in 1861 the population of England living in the cities and large towns amounted to 10,930,841; of those living in the country and in small towns, 9,134,386.

